# Linkage Replication for Chromosomal Region 13q32 in Schizophrenia: Evidence from a Brazilian Pilot Study on Early Onset Schizophrenia Families

**DOI:** 10.1371/journal.pone.0052262

**Published:** 2012-12-31

**Authors:** Ary Gadelha, Vanessa Kiyomi Ota, Jose Paya Cano, Maria Isabel Melaragno, Marilia A. C. Smith, Jair de Jesus Mari, Rodrigo A. Bressan, Sintia Iole Belangero, Gerome Breen

**Affiliations:** 1 Interdisciplinary Lab of Clinical Neurosciences (LiNC), and Schizophrenia Program (PROESQ), Department of Psychiatry, Universidade Federal de Sao Paulo (UNIFESP), São Paulo, Brazil; 2 Morphology and Genetics Department, Universidade Federal de Sao Paulo (UNIFESP), São Paulo, Brazil; 3 Medical Research Council Social Genetic and Developmental Psychiatry Centre, Institute of Psychiatry, King’s College London, London, United Kingdom; 4 National Institute of Health Research Biomedical Research Centre for Mental Health, Institute of Psychiatry, King’s College London, London, United Kingdom; University of Iowa Hospitals & Clinics, United States of America

## Abstract

We report analyses of a Brazilian study of early onset schizophrenia (BEOS) families. We genotyped 22 members of 4 families on a linkage SNP array and report here non-parametric linkage analyses using MERLIN® software. We found suggestive evidence for linkage on two chromosomal regions, 13q32 and 11p15.4. A LOD score of 2.71 was observed at 13q32 with a one LOD interval extending from 60.63–92.35 cM. From simulations, this LOD score gave a genome-wide empirical corrected p = 0.33, after accounting for all markers tested. Similarly 11p15.4 showed the same maximum LOD of 2.71 and a narrower one LOD interval of 4–14 cM. Of these, 13q32 has been reported to be linked to schizophrenia by multiple different studies. Thus, our study provides additional supporting evidence for an aetiological role of variants at 13q32 in schizophrenia.

## Introduction

Schizophrenia is a chronic, highly disabling disease that has a point prevalence estimate of ∼0.5% general population, which usually leads to persistent functional impairment with considerable morbidity and economic cost [1], [2]. It is considered a complex trait resulting from both genetic and shared environmental etiological influences. Several studies support a familial aggregation (as reviewed by Sullivan [3]) with a 10-fold increase in risk to sibling of one proband and up to a 40-fold increase when both parents are affected [4]. The genetic contribution to risk is high and heritability estimates based on clinical ascertainment are usually given as over 80% [5], [6]. A recent population-based study of 2 million families in Sweden put the estimate at a lower, but still considerable, 64.3%, with a 95% Confidence Interval (C.I.) estimate of 61·7%–67·5% [7].

Linkage analysis is a standard approach for identifying the location of genes that cause genetic diseases [8], whose primary advantage lies in detecting genes of moderate to major effect. This contrasts with genome-wide association studies, which are superior at finding loci of small effect [9]. Linkage studies conducted in schizophrenia have yielded positive findings in many different regions of the genome, with a somewhat confusing mixture of replication and non-replication. There is clear heterogeneity with no ‘hard’ replication for any region, with none being implicated in more than 20% independent studies [3].

Several reasons may explain these results, including locus and phenotypic heterogeneity, inadequate sample size, differences in ascertainment, marker sets, ancestry and statistical methods [10]. A systematic genome-scan meta-analysis [11] found 20 regions that achieved genome-wide linkage evidence. In a recent update of their meta-analysis that included 32 genome-wide SCZ studies, 10 regions were identified, with a concordant finding for the two meta-analysis on chromosome 2q (118.7–152 Mb) [10]. More specifically, they found that 10 genome bins are likely to contain loci linked to SCZ, including regions of chromosomes 1q, 2q, 3q, 4q, 5q, 8p and 10q. In a secondary analysis of 22 studies of European-ancestry samples, they reported suggestive evidence for linkage on chromosome 8p. Other loci have been reported and replicated in two regions of chromosome 13, one in the region of 13q21–33, (∼ 95 Mb) and another in the 13q13–14 region (∼ 40 Mb) [12]–[17].

In the present study we perform a pilot linkage study in Brazilian families with early-onset schizophrenia in Sao Paulo, Brazil. We report here the results of non-parametric linkage analyses in 4 small multiplex families.

## Methods

### Ethics Statement

This study was approved by Research Ethics Committee of UNIFESP [CEP No. 1737/06]. Written informed consent was obtained from all participants recruited or caregivers, on the behalf of minors/children, and, clinical and laboratory investigations were strictly conducted according to the principles expressed in the Declaration of Helsinki. All subjects invited accepted to enroll this study.

### Ascertainment of BEOS Families

22 subjects from 4 families, including 11 patients with schizophrenia and 1 with schizoaffective disorder were ascertained, yielding 8 affected sib-pairs and 6 other affected relative-pairs. All patients were being treated at the same specialized outpatient clinic in Sao Paulo, Brazil. Eligibility criteria were: one or more affected members with age of onset <18 years, and to have two or more members diagnosed with schizophrenia, excluding monozygotic twins. Exclusion criteria were comorbid mood disorders, mental retardation, and inability in providing informed consent.

### Clinical Assessment

The Structured Diagnostic Interview (SCID) was performed by trained psychiatrists to provide DSM-IV psychiatric diagnosis. Individuals under 18 years old were assessed with the Kiddie–Sads-Present and Lifetime Version [18]. The patients were also evaluated with the Positive and Negative Syndrome Scale (PANSS), Calgary Depression Scale [19], Clinical Global Impression (CGI), and Global Assessment of Functioning (GAF) instruments [20]. For the diagnosis, all available information, including medical record and family informant unstructured data, was used. When there was any doubt about the diagnosis, even for subtype, the interview and the medical records were reviewed by two other trained psychiatrists. There were 2 dubious cases, for subjects 41 and 42 in family 2. For subject 41 the doubt was whether best diagnosis would be schizoaffective disorder or schizophrenia. After careful consideration, we decided for schizoaffective disorder. For subject 42 the doubt was whether the number of symptoms and severity would allow the diagnosis of schizophrenia. After reevaluation we decided on the diagnosis of ‘psychosis not otherwise specified’.

### Clinical Demographics and Family Structures

**Figure 1:**
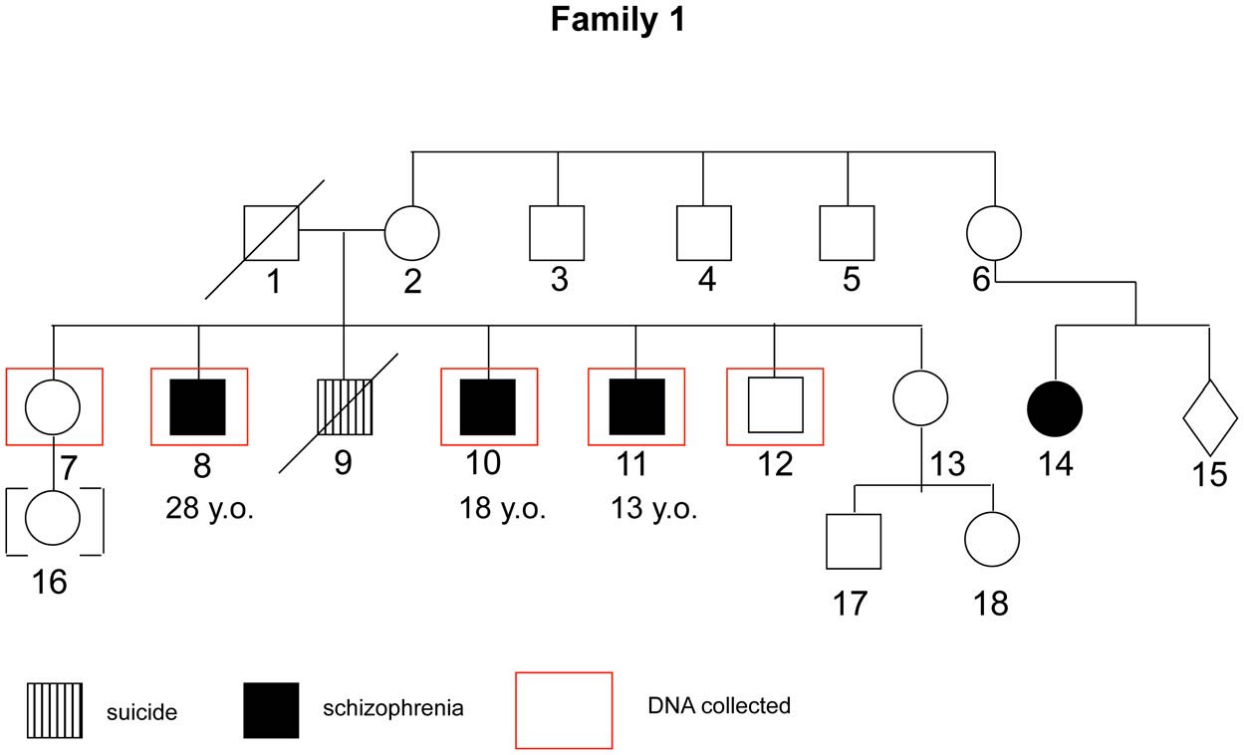
Family 1 - Three individuals affected with paranoid, treatment-resistant schizophrenia were enrolled. Father probably affected as reported by other family members but had no lifetime diagnosis. Another sibling, with a clear history of psychotic symptoms, committed suicide.

**Figure 2:**
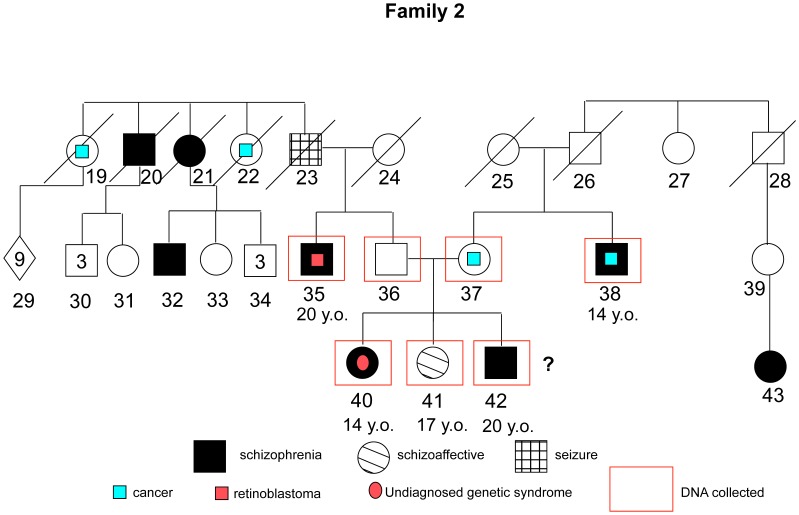
Family 2 - Five individuals affected enrolled: 2 paranoid, 1 hebephrenic, 1 schizoaffective and 1 with a DSM-IV diagnosis of psychosis not otherwise specified, probably an schyzotypal personality disorder. Both sides present history of schizophrenia over generations with no relation between them. In both sides there is also high incidence of cancers (retinoblastoma and brain tumors).

**Figure 3:**
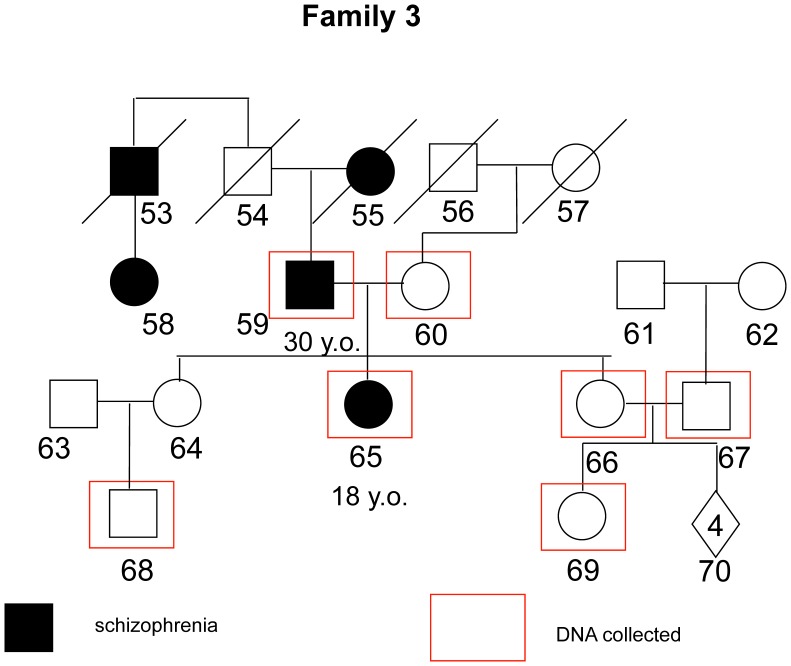
Family 3 - Two individuals affected enrolled, but clear evidence of other cases in previous generations. Both cases are paranoid schizophrenia, but with mild functional impairment and good response to antipsychotic treatment.

**Figure 4:**
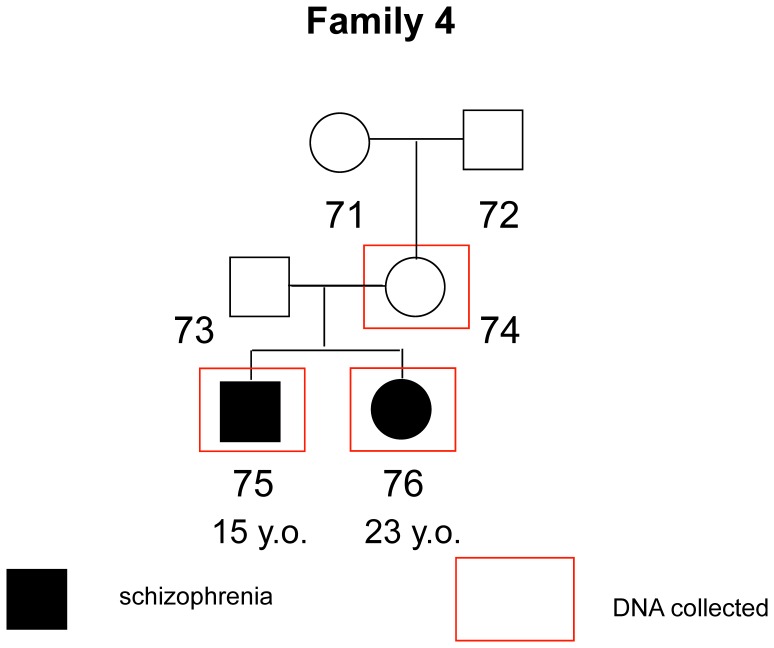
Family 4 - Both siblings affected with severe functioning impairment. Father probably affected but with no definite diagnosis and with no contact to the family.

The mean age of the whole sample was 41.4 (sd = 16.2); 43.5 (sd = 18.8) for non-affected individuals, and 39.7 (sd = 14.29) for patients. This difference was non-significant (Mann-Whitney U = 52.500; p = 0.62). For the whole sample, 54.5% are male and 45.5% are female; there was no significant difference in gender proportion between affected and non-affected individuals (Chi-square value = 1.56; p = 0.21). 7 out of 12 affected individuals presented onset age ≤18 years old, with a mean age of 15.57 (sd = 2.1). The 5 other affected subjects had a mean age of onset of 24.20 (sd = 4.6). A brief description of each family and the respective pedigree diagrams are provided in the figures below (Fig. 1–4):

### DNA Isolation and Quantification

Whole blood was collected into tubes containing 0.1% EDTA and genomic DNA isolation was performed using the Gentra™ Puregene™ Kit (Qiagen, Maryland, USA), according to the manufacturer’s protocol. DNA concentration was measured using a spectrophotometer (ND-1000 NanoDrop® Technologies, Wilmington, USA) and each sample was accurately quantified in triplicate and diluted to 100±0.5 ng/µL. Genotyping was performed using standard methods at laboratories at the MRC SGDP Centre, KCL. Briefly, Affymetrix 10 k arrays were typed in all samples and genotypes were called as per the manufacturer’s protocol.

### Family Linkage Analysis

All phenotypic information from interviews and questionnaires was coded and samples were assigned a number with removal of any personal identifying information. The program MERLIN [21] was used to perform non-parametric dichotomous linkage analysis. Map distances were provided by the annotation files supplied with the array. Allele frequencies were estimated using the maximum likelihood option in MERLIN using the entire sample. Merlin multipoint LOD scores were calculated at each marker and 10 positions between them. Specifically we used MERLIN’s –exp call to derive Whittemore and Halpern NPL pairs Z scores [22] under the exponential model, which allows for extreme allele sharing and which is more accurate in analyses of small collections of families. MERLIN converts these Z-scores into LOD scores using the method of [23]. Relationships were checked using the program GRR [24]. PEDSTATS [25] was used to detect mendelization errors. In addition the –error procedure was used to remove unlikely recombinants, while the –simulate procedure was used to, empirically, estimate the false positive rate (1000 simulations). Details of each family are show in figures 1–4.

## Results

### Linkage Results

**Figure 5:**
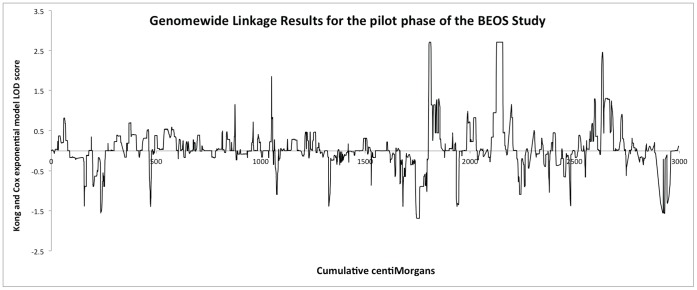
Genomewide linkage results for the BEOS study.

We found a suggestive linkage with a LOD score of 2.71 at 13q32 at 13q31/32 (Figure 5). The peak is broad with a one LOD interval extending from 60.63–92.35 cM (flanking markers rs723801 and and rs1323619). From simulations this LOD score gave a genome-wide empirical corrected p = 0.33 (uncorrected p = 0.0002062) accounting for all markers tested. Similarly 11p15.4 showed the same maximum LOD of 2.71 with a corrected p = 0.33 (uncorrected = 0.0002067) and a narrower one LOD interval of 4–14 cM (flanking markers rs720571 and rs2132517). One other region gave a LOD score >2 with a LOD of 2.45 on 19p13.3. The one-LOD interval spanned 5.6 and 11.8 cM but was supported by only 3 SNPs (rs2108389, rs1384936, rs639251) and did not exceed out suggestive linkage threshold derived from simulations of 2.57.

## Discussion

In our study we found suggestive evidence for linkage on chromosomal regions 13q32 and 11p15.4 under the criteria of Lander and Kruglyak [26]. The former region has already been implicated in several studies [OMIM SCZ7%603176]. Blouin et al. [13] reported a non-parametric linkage (NPL) analysis providing significant evidence for an SSL on chromosome 13q32 (NPL score = 4.18; p = 0.000002), and suggestive evidence for another SSL on chromosome 8p21–22 (NPL = 3.46; p = 0.0001). This replicated the earlier finding of a LOD score of 1.61 for marker D13S144 by Lin et al. [12]. Brzustowicz et al. [27] analyzed 21 Canadian families with schizophrenia and found genome-wide significant LOD scores on 13q. Brzustowicz et al. [28], using the same dataset and multipoint analysis and found a maximum LOD score of 3.81 with an empirical p = 0.02 under a recessive-broad model of schizophrenia at D13S793, with an estimated 65% of families linked to this region. Faraone et al. [15] assessed linkage between 13q and schizophrenia in 166 families. They found a suggestive maximum LOD score was 1.43 (Z-score of 2.57) at 79.0 cM with the peak being within 3 cM of the peak reported by Brzustowicz et al. [27]. Mulle et al. [29] reported a nonparametric linkage score in a sample of 29 sib-pairs with schizophrenia of 2.95 at D13S174, the same marker at which Blouin et al. [13] reported their maximum LOD.

Chromosome 11 includes several plausible candidate genes for major psychiatric disorders, especially psychosis, including the tyrosine hydroxylase (*TH*) and the dopamine receptor D4 (*DRD4*) (both at 11p15). However, the evidence for linkage in the region is weak. A review of the linkage results in the region concluded that they show a lack of replication but suggested that modest interest was maintained by possible signals scattered across a 30cM interval [30]. Some more recent studies found evidence for a role of chromosome 11p15.4 in bipolar disorder [31] autism [32] and attention deficit hyperactivity disorder [33].

This relative lack of previous evidence for chromosome 11p15 contrasted with the strong previous findings available for 13q32. Thus, although we should not disregard the linkage with 11p15.4, our primary finding is an additional evidence for a potential role for 13q32 region in a small Brazilian sample of families with early-onset schizophrenia affected individuals. The allele-sharing model tested is compatible with allelic homogeneity within the families. This region encompasses several genes, some of them already investigated in schizophrenia but with predominantly negative results like *ZIC2*, *SLC15A1*, and *FGF14*. Goes et al. [34] showed evidence for familial aggregation to mood incongruent psychotic symptoms among patients with bipolar disorder for 13q32 region. Other studies have reported association with the nearby 13q13–14 region (i.e., [17]). However, recent large schizophrenia GWAS analyses have failed to report significant association in both regions [35] and there are no obvious known variants that explain the observed linkage signal [10].

The major limitation of our study was the small sample size. However, families with early-onset schizophrenia were ascertained in an attempt to focus on a more severe and homogenous form of the illness. Likewise, although we find replication evidence for 13q32 and schizophrenia, the most recent meta-analysis [10] failed to yield significant results for this region, suggesting that there may be significant locus heterogeneity between studies and that, if this is a true linkage, then only a subset of families and cohorts show the effect. It should also be noted that this replication was not the primary aim of the study, which was exploratory. Another caveat relates to the probable genetic heterogeneity between families in regard to which individuals provided DNA. This could be an issue, but our primary method of analysis derives its information from the affected rather than the unaffected individuals.

Further analyses of family based early onset schizophrenia cohorts and large scale sequencing efforts may shed further light on the specific genetic factors influencing risk for schizophrenia in this region.
